# Habitat use of the white‐headed langurs in limestone forest of Southwest Guangxi, China: Seasonality and group size effects

**DOI:** 10.1002/ece3.9068

**Published:** 2022-07-04

**Authors:** Fengyan Liu, Youbang Li, Kechu Zhang, Jipeng Liang, Dengpan Nong, Zhonghao Huang

**Affiliations:** ^1^ Key Laboratory of Ecology of Rare and Endangered Species and Environmental Protection (Guangxi Normal University) Ministry of Education Guilin China; ^2^ Guangxi Key Laboratory of Rare and Endangered Animal Ecology Guangxi Normal University Guilin China; ^3^ Administration Center of Guangxi Chongzuo White‐headed Langur National Nature Reserve Chongzuo China

**Keywords:** food availability, group size, habitat use, predation risk, *Trachypithecus leucocephalus*

## Abstract

Understanding how animals cope with habitat‐specific environmental factors can assist in species conservation management. We studied the habitat use of four groups (two large and two small groups) of white‐headed langurs (*Trachypithecus leucocephalus*) living in the forest of southwest Guangxi, China between September 2016 and February 2017 via instantaneous scan sampling. Our results showed that the langurs primarily used hillsides (55.91% ± 6.47%), followed by cliffs (29.70% ± 5.48%), hilltops (7.26% ± 3.55%), flat zones (6.99% ± 6.58%), and farmlands (0.14% ± 0.28%). The langurs moved most frequently on hillsides (49.35% ± 6.97%) and cliffs (35.60% ± 9.17%). The hillsides were more frequently used (66.94% ± 7.86%) during feeding, and the langurs increased the use of hilltops during the rainy season, and the use of cliffs in the dry season. The langurs frequently rested on hillsides (49.75% ± 8.16%) and cliffs (38.93% ± 8.02%). The larger langur group used cliffs more frequently when moving and resting, whereas the small langur group used hillsides more frequently while resting. Langurs in all groups avoided the flat zones for feeding. Their use of habitat reflected the balancing of foraging needs, thermoregulation, and predator avoidance. We conclude that the ecological factors are determinants of habitat use for white‐headed langurs. Our findings suggest that conservation efforts should focus on protecting the vegetation on the hillsides and restoring the vegetation on the flat zones.

## Graphical abstract

Our findings underline the importance of these ecological factors on habitat use for white‐headed langurs and the need to understand how ecological seasonality and group sizes impact their behaviors in the limestone forest.

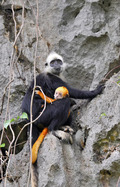

## 1 INTRODUCTION

Multiple factors affect habitat use by animals, including food availability (Bryson‐Morrison et al., 2017; Nagy‐Reis & Setz, 2017; Sargent et al., 2021), predation risk (Dickie et al., 2020; Jones et al., 2022; Monteza‐Moreno et al., 2020), and group size (Albani et al., 2020; Webber & Wal, 2021). Animals show flexibility in their behavioral responses to ecological fluctuation, and understanding how animals cope with habitat‐specific environmental factors can assist in species conservation management (Ni et al., 2018; Pennec et al., 2020).

Food resource availability significantly impacts habitat use in primates (Camaratta et al., 2017; Nagy‐Reis & Setz, 2017; Pennec et al., 2020). In general, food resources are spatiotemporally distributed, and primates prefer to forage in food‐abundant areas (Hongo et al., 2017; Ni et al., 2018). However, primates may choose to forage in more extensive home ranges to obtain adequate food intake when food resource availability declines (Nagy‐Reis & Setz, 2017). Moreover, they are even likely to forage in food‐rich areas with high‐predation risk due to low food availability elsewhere (Hendershott et al., 2018; Zhou et al., 2013). For example, Cat Ba langurs (*Trachypithecus poliocephalus*) forage in valleys (predatory‐risky) due to limited food resources in cliffs and hilltops (Hendershott et al., 2018). Similarly, Bale monkeys (*Chlorocebus djamdjamensis*) venture to steal cultivated foods in farmlands (predatory‐risky), supplementing their diets during low food availability periods (Mekonnen et al., 2020). Consequently, primates have to balance food and predation pressure in habitat use (Chen et al., 2019; Cowlishaw, 1997b; Huang et al., 2013).

Predation mortality is significant among primates (Cheney et al., 2004; Stanford et al., 1994), and primates keep vigilance to detect predator and to minimize predator attacks (Bolt et al., 2015; Matsumoto‐Oda et al., 2018; Stojan‐Dolar & Heymann, 2010). Normally, primates stay vigilant as much as possible, and several activities (i.e., resting and grooming) could decrease activity time spent on vigilance, forcing them to perform these activities in safe locations (Cords, 1995; Cowlishaw, 1997a; Maestripieri, 1993). Accordingly, primates prefer resting in tall trees or cliffs, which is easier for them to detect and/or avoid predator (Enstam & Isbell, 2004; Li et al., 2021). For example, resting Angolan colobus monkeys (*Colobus angolensis ruwenzorii*) stay in larger trees and at higher canopy heights rather than at lower canopy heights to decrease predation risk (Adams & Teichroeb, 2020).

Food availability influences primate group sizes (Chapman et al., 1995; Wrangham et al., 1993), and group sizes significantly affects travel distance/time as a reflection of intragroup feeding competition (Chapman, 1990; Liu et al., 2013; Zhang et al., 2020). Specifically, larger groups have greater energy requirements than smaller groups, making the former spend more time traveling and feeding to harvest adequate foods (Wang et al., 2021). Predation is another factor affecting primates' group sizes, and larger groups could decrease predation risk as they have an increase in predator detection and defense than smaller groups (Hill & Lee, 1998; Matsumoto‐Oda et al., 2018; Suscke et al., 2017). Consequently, for those primates inhabiting in smaller forest fragments than those inhabiting continuous forests (Ni et al., 2018), the larger primate groups are more likely to forage in food‐rich and high‐risk patches than smaller ones to obtain adequate foods (Adams & Teichroeb, 2020; Albani et al., 2020). In the South Sulawesi karst forest, larger groups of moor macaques (*Macaca Maura*) use food‐rich and high‐predation risk patches more frequently than small groups, which prefer areas with lower food availability but lower predation risk (Albani et al., 2020).

Fragmentation affects primate behaviors, habitat use, and foraging options (Huang et al., 2017; Mekonnen et al., 2018). The forest structure and composition in fragments may be altered, which possibly lead to the plant species diversity and abundance reduced (Arroyo‐Rodríguez & Mandujano, 2006; Mekonnen et al., 2017). As a result, primates living in small and isolated forest fragments may face challenges caused by the reduced food availability and smaller home‐range size (Huang et al., 2017; Mekonnen et al., 2018). Furthermore, primates living in fragments are likely to accept more anthropogenic disturbance and may avoid to utilize areas where human occur frequently (Waterman et al., 2019).

White‐headed langurs (*Trachypithecus leucocephalu*s) from southwest Guangxi, China, are distributed in a 200‐km^2^ area composed of degraded and fragmented forests (Huang, 2002; Huang, Li, et al., 2008). The small sizes of the fragments provide limited food species for these individuals (Huang et al., 2017). White‐headed langurs are extremely folivorous (Huang, 2002; Li et al., 2003) and prefer young leaves and fruits (Lu et al., 2021). However, the distribution of vegetation in limestone forests significantly differ in hill zones (Huang et al., 2002; Li & Rogers, 2006). According to the topography and the vegetation of the region, limestone hills can be roughly divided into five zones: hilltop, cliff, hillside, flat zone, and farmland (more details can be seen in the methods section) (Li & Rogers, 2006). Briefly, hilltops and cliffs have sparsely distributed vegetation and many bare rocks. The hillsides are covered by thick vegetation and lay below the cliffs. The flat zones have small amounts of plants, lying at the bottoms of the hills. The farmland is the part of flat zones and is used as agricultural areas. Moreover, since the variaitons in rainfall (with a distinct dry season and a rainy season), young leaves and fruits are seasonally abundant and unevenly distributed in the limestone forest (Huang, Wu, et al., 2008; Li et al., 2016; Zhou et al., 2006). Furthermore, group size makes different nutritional requirements for langur groups which do not have same group size (Zhang et al., 2020) Previous studies have showed that the habitat use of limestone‐living primates are usually associated with the distribution of food resource and predation risk (Huang et al., 2000;Li et al., 2021; Zhou et al., 2013). Terrestrial carnivores (e.g., leopard cat *Prionailurus bengalensis*) and raptors (e.g., crested goshawk *Accipiter trivirgatus*) in the limestone forests (Duan et al., 2020; Wu, 1983) are reported as significant death threats to infant primates (Huang, 2002). However, more information on how these factors (including food availability, group size and predation risk) affect the hill zones use by white‐headed langurs is unavailable.

In this study, our goals are to explore how the spatial distribution of food and seasonal variations, predation risk, and group size affect the habitat use pattern of white‐headed langurs. This may also provide critical information for this primate species conservation. We collected data on the individual habitat use patterns of white‐headed langurs and tested the following predictions:

1. We predict white‐headed langurs would mainly forage on the hillsides, and seasonally use other hill zones for foraging. White‐headed langurs are folivorous and prefer young leaves and fruits (Li et al., 2003; Lu et al., 2021). White‐headed langurs forage on the hillsides can harvest abundant foods, due to the fact that vegetation is densely distributed on the hillsides (Huang et al., 2002; Li & Rogers, 2006). Moreover, folivorous primates tend to look for preferred foods (young leaves and fruits) when their availability is higher during the rainy season (Chen et al., 2019).
2. We predict white‐headed langurs would use cliffs more frequently when resting. Primates resting on cliffs not only can more easily detect predators with a broader vision, but also can effectively prevent terrestrial carnivores approaching when resting (Li et al., 2021; Zhou et al., 2013), because cliffs in limestone forests are located in the upper part of the hills and are covered by sparse vegetation and consist of vertical bare rocks (Li & Rogers, 2006).
3. We predict white‐headed langurs both in large and in small groups would mainly forage on hillsides, and the langurs in large groups would expand foraging areas to flat zones for satisfying larger energy needs. White‐headed langurs in large groups have more intensive intragroup feeding competition than small groups (Zhang et al., 2020). Moreover, the vegetation on flat zones is relatively better compared with the cliffs and hilltops (Li & Rogers, 2006).

## 2 METHODS

### 2.1 Study site and study subjects

**FIGURE 1:**
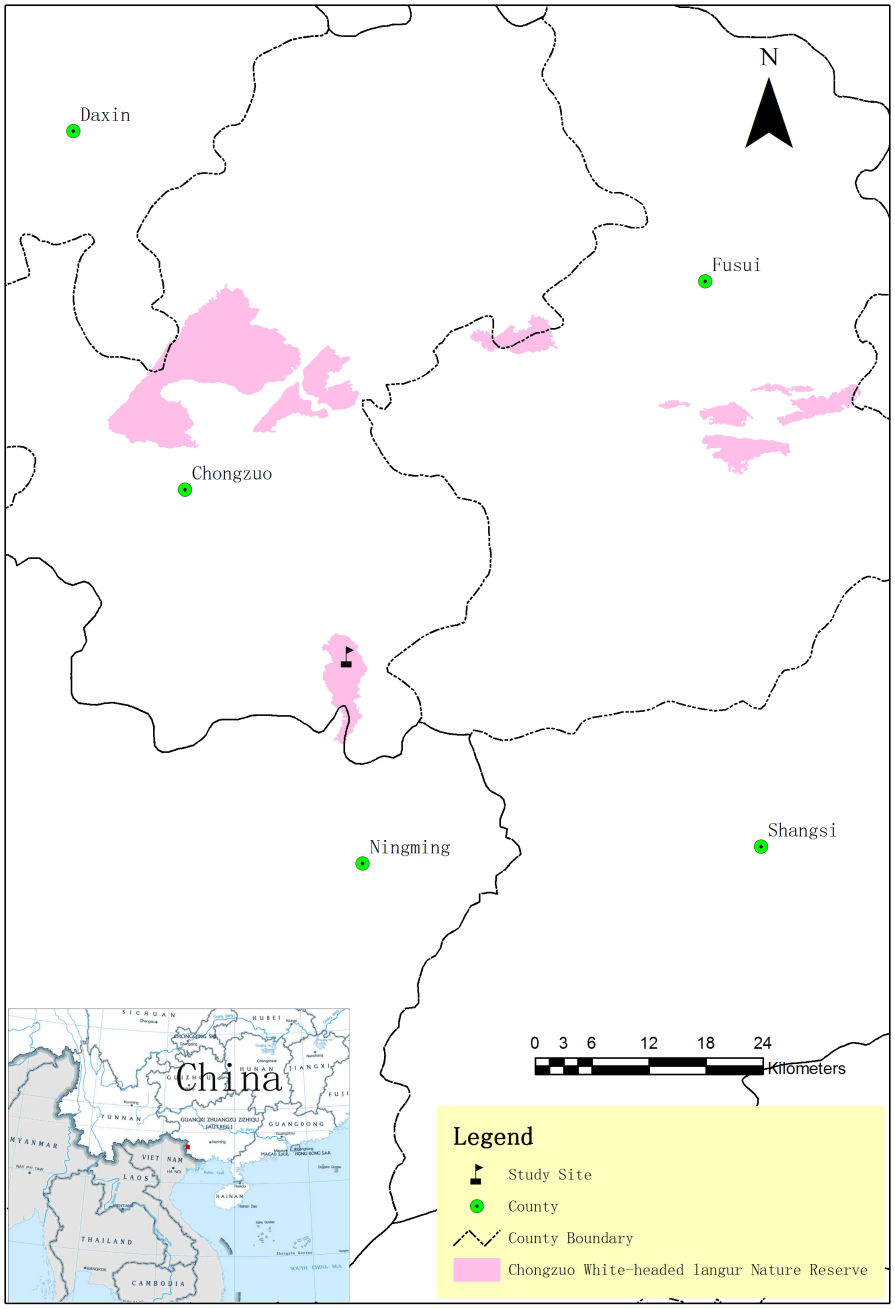
Location of the study site in Chongzuo white‐headed langur National Nature Reserve, Banli area, Southwest Guangxi, China (cited and modified from Huang et al. (2017))

This study was conducted between September 2016 and August 2017 in the Chongzuo White‐Headed Langur National Nature Reserve, southwest Guangxi, China (107°16′53″–107°59′46″E, 22°10′43″–22°36′55″N; Figure 1). The altitude of limestone hills ranges from 400 to 600 m, and vegetation is typical limestone seasonal rainforest (Guangxi Forestry Department, 1993). The habitat could be roughly divided into five zones considering the topography and the vegetation of the region: hilltop, cliff, hillside, flat zone, and farmland (Li & Rogers, 2006). The hilltops have bare rocks and sparse vegetation. The cliffs contain mainly bare vertical rocks and little vegetation, given the lack of soil and water. The hillsides are covered by thick vegetation with large trees, shrubs, and vines and lay below the cliffs. The flat zones are covered by small amounts of trees and shrubs due to historical cultivation, lying at the bottoms of the hills. Farmland is part of flat zones and is used for agriculture by local residents. We did not assess the availability of each hill zone in the habitat, and made records based on observed use pattern by langurs.

Climatic factors were collected during the study period using an automatic electronic thermometer and a rain gauge. We set one thermometer in the middle forest layer, and one on bare rock to record temperatures. The mean annual temperature of bare rock was 26.3°C, and the mean temperature of forest was 22.5°C. Zhang et al. (2021) shows more detailed temperature data (e.g., mean highest/lowest temperature of forest and bare rock). The annual precipitation was 4382.9 mm (Table 1). Based on the precipitation of the region, the study period was roughly divided into dry (September 2016 to February 2017) and rainy (March to August 2017) seasons (Zhang et al., 2020).

**TABLE 1:** Group size, number of days sampled and scans recorded for the langur groups, and rainfall records

Month	Group size				Full‐day sample days/total sample days				Total scans				Rainfall, mm
	G‐DS	G‐ZWY	G‐LZ	G‐NN	G‐DS	G‐ZWY	G‐LZ	G‐NN	G‐DS	G‐ZWY	G‐LZ	G‐NN	
*2016*													
September	15	16	6	5	2/5	3/3	3/5	3/3	101	116	165	147	84.4
October	15	18	6	5	4/4	4/4	5/6	4/5	153	177	189	207	385.0
November	16	18	6	5	4/4	4/5	4/5	5/5	113	117	177	179	126.5
December	20	18	6	5	5/5	5/5	4/5	5/5	217	169	185	216	37.0
*2017*													
January	21	19	6	5	4/5	5/5	5/5	5/5	165	174	153	200	240.0
February	24	19	6	6	5/5	5/5	5/5	5/5	201	201	205	232	135.0
March	24	19	7	8	4/5	4/5	4/4	5/5	187	158	183	232	434.0
April	24	19	7	8	5/5	5/5	5/5	5/5	155	190	221	259	77.0
May	24	19	6	9	5/5	4/5	5/5	5/5	222	208	270	258	840.0
June	24	19	7	9	4/5	4/5	5/5	5/5	224	229	237	259	690.0
July	25	19	8	9	5/5	4/5	5/5	5/5	217	231	281	267	567.0
August	25	19	8	9	5/5	5/5	5/5	5/5	246	253	268	259	767.0
Total					52/58	52/57	55/60	57/58	2201	2223	2534	2715	4382.9
Mean					4.3/4.8	4.3/4.8	4.6/5	4.75/4.8	183.4	185.3	221.2	226.3	365.2
SD					0.9/0.4	0.7/0.6	0.7/0.4	0.6/0.58	46.2	42.7	43.7	37.7	291.3
Dry season					24/28	26/27	26/31	27/28	950	954	1074	1181	1007.9
Mean					4/4.7	4.3/4.5	4.3/5.2	4.5/4.7	158.3	159	179	196.8	168.0
SD					1.1/0.5	0.82/0.83	0.8/0.4	0.84/0.82	46.2	34.7	18.4	30.1	125.9
Rainy season					28/30	26/30	29/29	30/30	1251	1269	1460	1534	3375.0
Mean					4.7/5	4.3/5	4.8/4.8	5/5	208.5	211.5	243.3	255.7	562.5
SD					0.5/0	0.5/0	0.4/0.4	0/0	32.3	33.9	37.2	12.1	278.3

The nature reserve consists of four parts, Bapen, Banli, Tuozhu, and Dalin, and 44 groups of langurs lives in the Banli part (Figure 1), including approximately 450 individuals (~10.2 members per group; Zhang et al., 2020). Behavioral data from four of these groups were collected. At the beginning of the study, the G‐DS group included 15 individuals (adult male: adult female: subadult 1:13:1), the G‐ZWY group contained 16 individuals (adult male: adult female: infant 1:9:6), the G‐LZ group had six langurs (adult male: adult female 1:5), the G‐NN group had five individuals (adult male: adult female 1:4) The G‐DS and G‐ZWY groups were classified as large, and the G‐LZ and G‐NN groups were classified as small. Detailed descriptions of the groups are shown in Table 1.

### 2.2 Vegetation composition

We randomly sampled 37 plots (30 plots: 20 m × 20 m; 7 plots: 10 m × 10 m) across the main study site (including flat zones, hillsides, and hilltops) to investigate the vegetation composition. Specifically, 9 plots were on the flat zones, 16 plots were on the hillsides, and 12 plots were on the hilltops. We did not set up plots on the cliffs and farmlands because cliffs were inaccessible and farmlands were agricultural areas. We recorded and identified trees, shrubs, and woody lianas with diameter at breast height (DBH) or basal diameter >2 cm within plots (the diameter at approximately 1.2 m from the ground as DBH for woody lianas). We recorded species canopy height and width to calculate canopy volume. Following (Hu, 2011), we used basal area as an index of the leaf biomass of each plant and summed the cumulative leave biomass for per species in specify hill zones.

### 2.3 Behavioral data collection

The full‐day observations of the langurs began after their sleeping sites were located at dawn and ended at sunset. The data sampling in the partial‐day observations of the langurs began whenever they were observed and ended when the contact with the group was lost for more than 30 min or when they entered a sleeping site (Huang et al., 2017). During the study period, 216 full‐day observations were obtained (G‐DS: 52 days; G‐ZWY: 52 days; G‐LZ: 55 days; G‐NN: 57 days) (Table 1).

The behavioral data on the white‐headed langurs were collected via instantaneous scan sampling (Altmann, 1974). We set 15 min for one scan unit. More specifically, the first 5 min of each scan were used for behavioral sampling, followed by a 10‐min interval until the following scan began. The group numbers were scanned from left to right or clockwise to avoid sampling bias toward specific individuals. We collected behavioral data of as many individuals as possible during each scan. No individuals were recorded twice. In each scan mission, the location (i.e., hilltop, cliff, hillside, flat zone, and farmland) of the scanned individuals and their predominant behavior (resting, moving, and feeding; Huang et al., 2017) were recorded after observing them for 5 sec. We defined the location and predominant behavior of focal group during each scan according to the location and predominant behavior that occurs in the majority of individuals (Chen et al., 2019). Infants were excluded from scanning, because their behaviors were not independent. When feeding, the plant species and part eaten by the langurs were recorded. A total of 9673 scans were obtained, composed of 2201 (G‐DS); 2223 (G‐ZWY); 2534 (G‐LZ); and 2715 (G‐NN) scans (Table 1).

### 2.4 Data analysis

We set each scan of group as one independent record and determined the monthly utilization percentage for different hill zones by calculating the frequency of specific hill zones in the monthly total records. The annual and seasonal utilization frequencies were expressed as the average values of relevant months (Chen et al., 2019). A similar method was used to calculate the annual and seasonal utilization frequency across behaviors. We calculated the canopy volume (m^3^/ha) for each species within plots and determined the species canopy volume in different hill zones (including flat zone, hillside, and hilltop) by calculating the canopy volume of all species in all plots in specific region. Moreover, based on the feeding records of plant species by the langurs, the density (individual/ha), basal area (m^2^/ha), and canopy volume of food species in different hill zones were calculated. Spearman's rank correlation was performed to test the correlation between species canopy volume and utilized frequency of specific hill zone, as well as was used to detect the correlation between distribution density, basal area, and canopy volume of food species and utilized frequency of each hill zone.

A Kruskal–Wallis test was used to evaluate the difference between multiple independent samples regarding whether the white‐headed langurs had a difference in habitat use, and to detect whether the density, basal area and canopy volume of food species in these hill zones are different. Following Zhang et al. (2020), generalized linear mixed models were performed to examine the season and group size influences on habitat use. Specifically, the utilization frequency of hill zones was treated as response variable, the seasons were set as fixed factors, and the group was set as a random factor to examine the seasonal differences in habitat use. Similarly, the group was set as a fixed factor, and the season was set as random factors to examine the effect of group size on habitat use. Season or group size were considered key factors when they influenced the goodness‐of‐fit of the model when the *p*‐value was lower than .05, indicating a significant difference in habitat use between the dry and rainy seasons or between large and small groups (Kurihara & Hanya, 2015; Zhang et al., 2020). Generalized linear mixed models were performed with the *lime4* package in R v.4.0.4 (R Core Team, 2021). All variables expressed as percentages were logit‐transformed to improve the linearity and normality of the test (Warton & Hui, 2011).

## 3 RESULTS

### 3.1 The relationship between vegetation composition and hill zones use

Species canopy volume on hillsides was highest (5.41 × 10^−4^ m^3^/ha), followed by flat zones (3.95 × 10^−4^ m^3^/ha) and hilltops (1.06 × 10^−4^ m^3^/ha). Species canopy volume was positively correlated with the overall utilized frequency of hill zones (*r*_*s*_ = 0.450, *n* = 20, *p =* .047), and with the utilized frequency of hill zones when feeding (*r*_*s*_ = 0.612, *n* = 20, *p =* .004). However, no significant correlations were detected between the species canopy volume and utilized frequency of hill zones when moving or resting behaviors occurred (moving: *r*_*s*_ = 0.379, *n* = 20, *p =* .099; resting: *r*_*s*_ = 0.169, *n* = 20, *p =* .475).

The density, basal area, and canopy volume of food species in these hill zones are significantly different (density, *χ* ^2^ = 56.796, *p* < .001, *df* = 2; basal area, *χ* ^2^ = 29.901, *p* < .001, *df* = 2; canopy volume, *χ* ^2^ = 73.267, *p* < .001, *df* = 2). The density of food species on hilltops was highest (2.07 × 10^−5^/ha ± 6.90 × 10^−6^/ha, Mean ± SD), followed by hillsides (1.95 × 10^−5^/ha ± 3.81 × 10^−6^/ha), and flat zones (1.24 × 10^−5^/ha ± 3.78 × 10^−6^/ha). The highest basal area (9.51 × 10^−8^ m^2^/ha ± 7.80 × 10^−8^ m^2^/ha) and canopy volume (8.86 × 10^−5^ m^3^/ha ± 3.43 × 10^−5^ m^3^/ha) of food species was on hillsides, followed by flat zones (basal area: 5.71 × 10^−8^ m^2^/ha ± 5.30 × 10^−8^ m^2^/ha; canopy volume: 6.51 × 10^−5^ m^3^/ha ± 5.43 × 10^−5^ m^3^/ha) and hilltops (basal area: 3.42 × 10^−8^ m^2^/ha ± 2.36 × 10^−8^ m^2^/ha; canopy volume: 2.12 × 10^−5^ m^3^/ha ± 1.32 × 10^−5^ m^3^/ha).

The density, basal area, and canopy volume of food species on hilltops were positively correlated with the langurs use of hilltops across behaviors; density of food species is correlated with moving and feeding on hilltops (Table 2). Moreover, positive correlations were detected between the density of food species on hillsides and the utilized frequency of hillsides across behaviors. The basal area of food species on hillsides had a positive correlation with the utilized frequency of hillsides when feeding (Table 2). The basal area of food species on flat zones was positively correlated with the utilized frequency of flat zones when feeding. Similarly, significant correlations were found between the canopy volume of food species on flat zones and the utilized frequency of flat zones (Table 2).

**TABLE 2:** Correlations between the density (individual/ha), basal area (m^2^/ha), canopy volume (m^3^/ha) of food species and percentage of scan groups spent in each hill zone (including hilltops, hillsides, and flat zones)

Food species on each hill zone	Utilized frequency of each hill zone			
	Overall	Moving	Feeding	Resting
*Hilltops*				
Density	0.361*	0.287*	0.305*	0.219
Basal area	0.480**	0.421**	0.362*	0.332*
Canopy volume	0.459**	0.508**	0.298*	0.314*
*Hillsides*				
Density	0.512**	0.417**	0.566**	0.373
Basal area	0.197	0.075	0.302*	0.103
Canopy volume	−0.215	−0.274	−0.069	−0.232
*Flat zones*				
Density	0.182	0.052	0.236	0.040
Basal area	0.231	0.162	0.312*	0.106
Canopy volume	0.706**	0.624**	0.780**	0.549**

* *p* < .05

** *p* < .01.

### 3.2 The overall hill zones use pattern

Significant differences were detected in the use of different hill zones by white‐headed langurs (G‐DS, *χ* ^2^ = 53.768, *p* < .001, *df* = 4; G‐ZWY, *χ* ^2^ = 50.957, *p* < .001, *df* = 4; G‐LZ, *χ* ^2^ = 47.620, *p* < .001, *df* = 4; G‐NN, *χ* ^2^ = 51.292, *p* < .001, *df* = 4). Hillsides was the most commonly used zone by the langurs (55.91% ± 6.47% of total records of the four groups, Mean ± SD), followed by cliffs (29.70% ± 5.48%), hilltops (7.26% ± 3.55%), flat zones (6.99% ± 6.58%), and farmlands (0.14% ± 0.28%) (Table 3).

**TABLE 3:** Limestone hill zones used by white‐headed langurs (%, means ± SD)

Activity pattern	Group	Hill zones					Kruskal–Wallis test	
		Hilltop	Cliff	Hillside	Flat zone	Farmland	*χ* ^2^(*df*=4)	*p*
Overall	G‐DS	10.77 ± 3.93	36.93 ± 14.50	51.00 ± 14.45	1.29 ± 1.97	0	53.768	<.001
	G‐ZWY	2.33 ± 1.99	30.94 ± 15.04	50.28 ± 18.63	15.88 ± 6.55	0.57 ± 0.96	50.957	<.001
	G‐LZ	8.16 ± 2.87	25.19 ± 17.34	63.85 ± 19.01	2.81 ± 5.36	0	47.620	<.001
	G‐NN	7.79 ± 2.93	25.72 ± 12.80	58.51 ± 11.54	7.97 ± 4.00	0	51.292	<.001
	Mean	7.26 ± 3.55	29.70 ± 5.48	55.91 ± 6.47	6.99 ± 6.58	0.14 ± 0.28		
Moving	G‐DS	10.22 ± 5.03	45.25 ± 9.10	43.61 ± 10.62	0.92 ± 1.67	0	53.294	<.001
	G‐ZWY	1.87 ± 1.81	40.18 ± 15.93	43.13 ± 21.48	13.39 ± 8.31	1.43 ± 2.29	44.691	<.001
	G‐LZ	8.23 ± 6.81	32.73 ± 21.77	56.43 ± 18.08	2.60 ± 3.95	0	47.620	<.001
	G‐NN	14.33 ± 9.32	24.21 ± 8.42	54.24 ± 11.33	7.22 ± 4.62	0	50.692	<.001
	Mean	8.66 ± 5.19	35.60 ± 9.17	49.35 ± 6.97	6.03 ± 5.58	0.36 ± 0.72		
Feeding	G‐DS	6.98 ± 6.00	21.23 ± 20.92	69.03 ± 21.33	2.76 ± 3.91	0	47.129	<.001
	G‐ZWY	2.80 ± 3.10	8.50 ± 8.38	56.57 ± 18.41	31.54 ± 14.22	0.58 ± 1.59	47.822	<.001
	G‐LZ	8.69 ± 6.85	12.44 ± 16.51	75.50 ± 17.58	3.38 ± 5.01	0	42.498	<.001
	G‐NN	4.50 ± 3.23	15.71 ± 12.72	66.67 ± 11.65	13.12 ± 4.68	0	49.619	<.001
	Mean	5.74 ± 2.61	14.47 ± 5.38	66.94 ± 7.86	12.70 ± 13.43	0.15 ± 0.29		
Resting	G‐DS	14.21 ± 5.06	45.98 ± 16.90	39.63 ± 16.35	0.19 ± 0.66	0	53.010	<.001
	G‐ZWY	2.20 ± 3.18	45.76 ± 23.12	47.18 ± 24.21	4.86 ± 5.52	0	48.953	<.001
	G‐LZ	7.69 ± 3.04	31.63 ± 17.06	58.36 ± 20.66	2.32 ± 6.04	0	51.870	<.001
	G‐NN	8.37 ± 3.67	32.36 ± 16.08	53.84 ± 15.00	5.43 ± 6.75	0	50.901	<.001
	Mean	8.12 ± 4.91	38.93 ± 8.02	49.75 ± 8.16	3.20 ± 2.42	0		

There were significant differences on the use of hill zones with specific behaviors (Table 3). White‐headed langurs more frequently used hillsides (49.35% ± 6.97%) and cliffs (35.60% ± 9.17%) while moving, followed by hilltops (8.66% ± 5.19%) and flat zones (6.03% ± 5.58%). While feeding, the langurs used hillsides most frequently (66.94% ± 7.86%), and they spent less time feeding on the cliffs (14.47% ± 5.38%), flat zones (12.70% ± 13.43%), and hilltops (5.74% ± 2.61%). Only individuals from the G‐ZWY group went onto farmlands (moving: 1.43% ± 2.29%; feeding: 0.58% ± 1.59%). When resting, langurs occurred more frequently on hillsides (49.75% ± 8.16%) and cliffs (38.93% ± 8.02%), followed by hilltops (8.12% ± 4.91%) and flat zones (3.20% ± 2.42%).

### 3.3 Seasonal hill zones use variations

**FIGURE 2:**
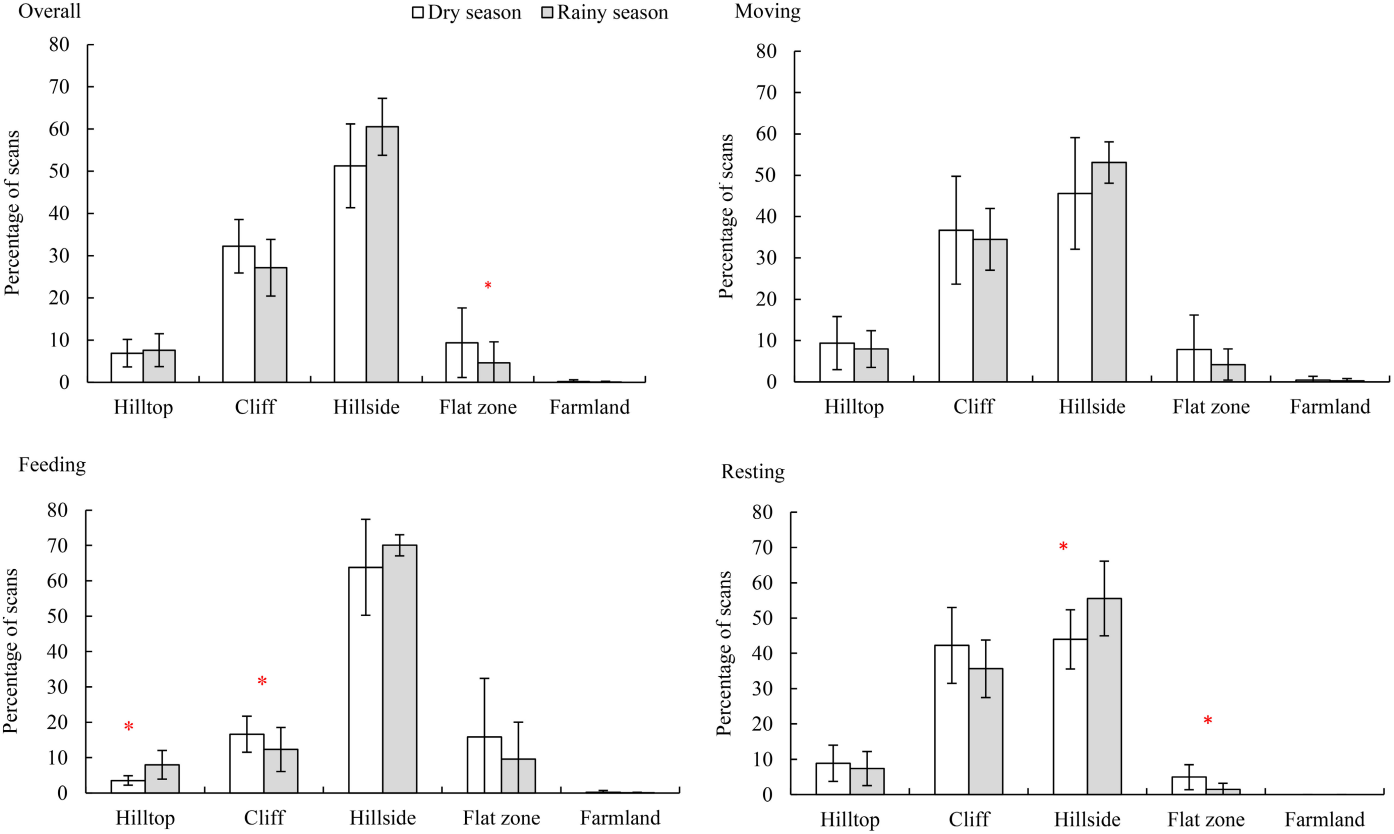
Seasonal variations in hill zones use (overall, moving, feeding and resting) of white‐headed langurs in limestone forest of Southwest Guangxi, China. Asterisks represent statistically significant differences between the dry and rainy season

Seasonal variations were not detected for most hill zones use by white‐headed langurs, except for the flat zones (Table 4 and Figure 2). These langurs exploited the flat zones more frequently during the dry season than during the rainy season (9.37% ± 8.26% vs. 4.61% ± 4.97%, *χ* ^2^ = 6.121, *df* = 1, *p* = .013).

**TABLE 4:** Comparisons of utilization frequency on hill zones for white‐headed langurs: Effect of season

Activity pattern	Response variable	Explanatory variable	Estimate	*SE*	*t*	*χ* ^2^ (*df* = 1)	*p*
Overall	Hilltop	Intercept	−1.358	0.249	−5.443	1.166	.280
		Rainy season	0.164	0.153	1.075		
	Cliff	Intercept	−0.361	0.081	−4.427	1.640	.200
		Rainy season	−0.122	0.095	−1.278		
	Hillside	Intercept	0.025	0.077	0.321	3.789	.052
		Rainy season	0.171	0.087	1.966		
	Flat zone	Intercept	−1.621	0.676	−2.397	6.121	.013*
		Rainy season	−0.875	0.345	−2.533		
	Farmland	Intercept	−4.605	0.289	−15.918	1.307	.253
		Rainy season	−0.258	0.227	−1.139		
Moving	Hilltop	Intercept	−1.807	0.500	−3.612	0.042	.837
		Rainy season	0.085	0.419	0.203		
	Cliff	Intercept	−0.270	0.107	−2.534	0.009	.924
		Rainy season	−0.010	0.103	−0.095		
	Hillside	Intercept	−0.252	0.170	−1.481	1.613	.204
		Rainy season	0.284	0.224	1.266		
	Flat zone	Intercept	−2.317	0.798	−2.904	0.571	.450
		Rainy season	−0.304	0.406	−0.749		
	Farmland	Intercept	−4.555	0.329	−13.860	1.244	.265
		Rainy season	−0.286	0.258	−1.110		
Feeding	Hilltop	Intercept	−2.289	0.329	−6.965	6.273	.012*
		Rainy season	0.930	0.362	2.567		
	Cliff	Intercept	−0.856	0.227	−3.769	4.451	.035*
		Rainy season	−0.661	0.312	−2.123		
	Hillside	Intercept	0.293	0.108	2.716	1.169	.280
		Rainy season	0.121	0.113	1.076		
	Flat zone	Intercept	−1.499	0.763	−1.964	3.731	.053
		Rainy season	−0.761	0.390	−1.951		
	Farmland	Intercept	−4.843	0.176	−27.546	0.014	.907
		Rainy season	−0.023	0.199	−0.115		
Resting	Hilltop	Intercept	−1.523	0.455	−3.347	0.560	.454
		Rainy season	0.187	0.251	0.742		
	Cliff	Intercept	−0.154	0.100	−1.546	1.739	.187
		Rainy season	−0.146	0.111	−1.317		
	Hillside	Intercept	−0.139	0.097	−1.437	4.526	.033*
		Rainy season	0.243	0.113	2.158		
	Flat zone	Intercept	−2.560	0.762	−3.357	4.430	.035*
		Rainy season	−0.760	0.356	−2.134		

* *p* < .05.

There were marked seasonal variations in the use of different hill zones during the feeding and resting periods of the langurs, but not during their moving (Table 4 and Figure 2). Specifically, the langurs used hilltops for feeding more frequently in the rainy season (7.97% ± 4.03% vs. 3.52% ± 1.31%, *χ* ^2^ = 6.273, *df* = 1, *p* = .012) and cliffs in the dry season (16.63% ± 5.09% vs. 12.31% ± 6.25%, *χ* ^2^ = 4.451, *df* = 1, *p* = .035). There was no pronounced seasonal variation in how often the langurs used the other hill zones when feeding. The langurs used hillsides for resting more frequently in the rainy season (55.55% ± 10.61% vs. 43.95% ± 8.40%, *χ* ^2^ = 4.430, *df* = 1, *p* = .033) and flat zones in the dry season (4.95% ± 3.56% vs. 1.44% ± 1.70%, *χ* ^2^ = 4.430, *df* = 1, *p* = .035). For the rest of hill zones, there was no significantly seasonal variation in use frequency when resting. When moving, the langurs had no significantly seasonal changes in the hill zones use (Table 4; Figure 2).

### 3.4 Group size effect on hill zones use

**FIGURE 3:**
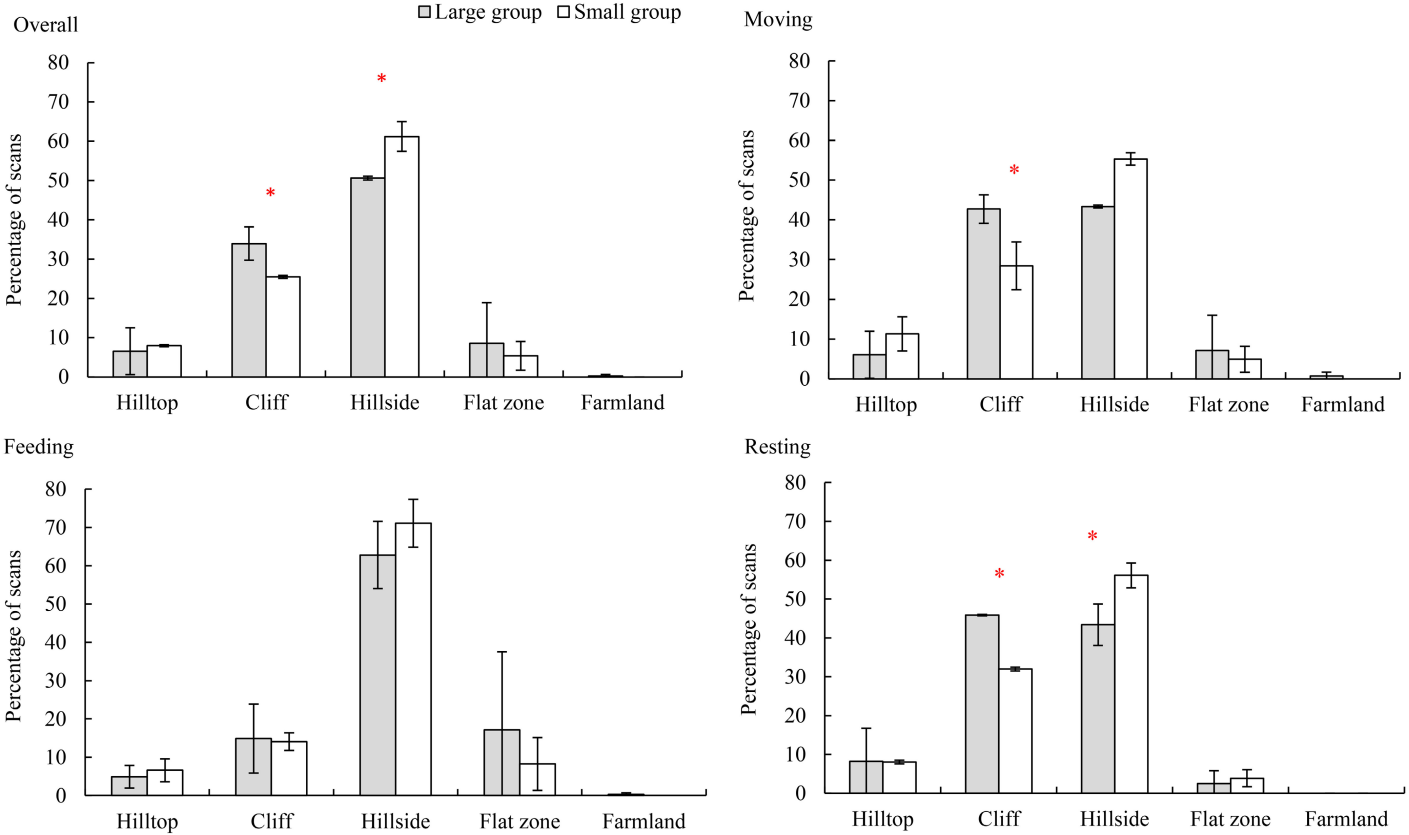
Difference in hill zones use (overall, moving, feeding and resting) for the large and small groups of white‐headed langurs in limestone forest of Southwest Guangxi, China. Asterisks represent statistically significant differences between the large and small groups

There were significant variations in use frequencies for cliffs and hillsides between the large and small groups (Table 5 and Figure 3). The large groups used cliffs more commonly (33.94% ± 4.24% vs. 25.45% ± 0.38%, *χ* ^2^ = 4.375, *df* = 1, *p* = .036), whereas the small groups used hillsides (61.18% ± 3.77% vs. 50.64% ± 0.51%, *χ* ^2^ = 4.929, *df* = 1, *p* = .026) more frequently. However, the langurs, whether in large or small groups, did not use any other hill zones differently.

**TABLE 5:** Comparisons of utilization frequency on hill zones for white‐headed langurs: Effects of group size

Activity pattern	Response variable	Explanatory variable	Estimate	*SE*	*t*	*χ* ^2^ (*df* = 1)	*p*
Overall	Hilltop	Intercept	−1.461	0.367	−3.980	0.910	.340
		Small group	0.371	0.519	0.715		
	Cliff	Intercept	−0.319	0.067	−4.746	4.375	.036*
		Small group	−0.205	0.095	−2.157		
	Hillside	Intercept	0.008	0.063	0.127	4.929	.026*
		Small group	0.204	0.089	2.309		
	Flat zone	Intercept	−2.062	1.132	−1.821	0	.995
		Small group	0.007	1.602	0.004		
	Farmland	Intercept	−4.468	0.376	−11.870	1.622	.203
		Small group	−0.532	0.532	−1.000		
Moving	Hilltop	Intercept	−1.972	0.760	−2.597	0.287	.592
		Small group	0.414	1.074	0.386		
	Cliff	Intercept	−0.137	0.085	−1.620	4.963	.026*
		Small group	−0.276	0.120	−2.304		
	Hillside	Intercept	−0.294	0.158	−1.858	2.740	.098
		Small group	0.367	0.224	1.644		
	Flat zone	Intercept	−2.494	1.336	−1.867	0.002	.969
		Small group	0.052	1.889	0.028		
	Farmland	Intercept	−4.395	0.428	−10.280	1.622	.203
		Small group	−0.605	0.605	−1.000		
Feeding	Hilltop	Intercept	−2.086	0.396	−5.265	1.450	.229
		Small group	0.524	0.560	0.935		
	Cliff	Intercept	−1.117	0.278	−4.021	0.190	.663
		Small group	−0.140	0.393	−0.355		
	Hillside	Intercept	0.263	0.131	2.005	1.561	.212
		Small group	0.181	0.186	0.977		
	Flat zone	Intercept	−1.768	1.273	−1.389	0.030	.862
		Small group	−0.222	1.800	−0.123		
	Farmland	Intercept	−4.710	0.205	−22.950	1.622	.203
		Small group	−0.290	0.290	−1.000		
Resting	Hilltop	Intercept	−1.766	0.679	−2.602	0.878	.349
		Small group	0.672	0.960	0.701		
	Cliff	Intercept	−0.084	0.077	−1.082	6.167	.013*
		Small group	−0.287	0.110	−2.623		
	Hillside	Intercept	−0.144	0.082	−1.757	4.559	.033*
		Small group	0.253	0.116	2.184		
	Flat zone	Intercept	−3.099	1.274	−2.433	0.062	.803
		Small group	0.320	1.802	0.177		

* *p* < .05.

While feeding, large and small groups did not significantly differ in their hill zones use, and both groups primarily foraged on hillsides. When resting behaviors occurred, the large groups used cliffs more commonly (45.87% ± 0.15% vs. 32.00% ± 0.52%, *χ* ^2^ = 6.167, *df* = 1, *p* = .013), whereas the small groups used hillsides more frequently (56.10% ± 3.20% vs. 43.40% ± 5.34%, *χ* ^2^ = 4.559, *df* = 1, *p* = .033). Moreover, large groups spent more time moving on cliffs (42.72% ± 3.58% vs. 28.47% ± 6.03%, *χ* ^2^ = 4.963, *df* = 1, *p* = .026). There were no differences between the large and small groups when using the other hill zones as resting or moving areas (Table 5 and Figure 3).

## 4 DISCUSSION

White‐headed langurs primarily foraged on the hillsides, and foraged less frequently on the cliffs/flat zones, supporting prediction 1 that white‐headed langurs would mainly forage in hillsides, and seasonally use other hill zones for foraging. Primates are biased toward food resource‐abundant areas when foraging (Albert et al., 2013; Terada et al., 2015), as shown in the current study. White‐headed langurs are highly folivorous (Huang, 2002). Results of the vegetation survey in our study are consistent with previous findings that food species distributed on the hillsides could provide abundant food resources for langurs (Li & Rogers, 2006; Zhou et al., 2013). Furthermore, the canopy volume of all plant species on the hillsides is highest, and dense and high canopy offers primates more predation protection with more escape routes and concealment sites (Adams & Teichroeb, 2020; Madden et al., 2010). Therefore, hillsides are food‐rich low‐risk areas for white‐headed langurs, and feeding on the hillsides assists them in safely harvesting adequate foods. The flat zones were used less frequently, likely because some are cultivated zones (Huang et al., 2002; Li & Rogers, 2005). Common human activities (i.e., sugarcane cultivation) prevented the langurs from using those zones for foraging. Given the illegal hunting on langurs commonly occurs in the past (Huang et al., 2002; Wang et al., 2005), the langurs generally avoid to utilize areas with high human activities and/or utilize these areas only when human disappear (Waterman et al., 2019).

The langurs spent more time foraging on the hilltops during the rainy season, probably because their preferred food items (i.e., young leaves and fruits) are available in these areas. Food resource distribution varies spatiotemporally (Chen et al., 2019; Hendershott et al., 2018). The langurs spent more time foraging on the hilltops during the rainy season, probably because their preferred food items (young leaves and fruits; Li & Rogers, 2006; Lu et al., 2021) are more available at this time (Zheng et al., 2021). Sympatric Assamese macaques (*M. assamensis*) show similar trends linked to the temporally increase in availability of hilltop fruits (Li et al., 2017). Contrarily, white‐headed langurs used cliffs more frequently during the dry season than during the rainy season when feeding, a trend that was probably related to the reduced availability of young leaves and fruits during the dry season. White‐headed langurs consume more mature leaves during the dry season (Li et al., 2016), and the consumption of mature leaves is negatively correlated with young leaves and fruits availability (Lu et al., 2021). In limestone forests, mature leaves are available year round and are distributed throughout the vegetation (Li et al., 2016; Li & Rogers, 2006), likely allowing the langurs to harvest less‐preferred food items in a wider‐ranger of hill zones, including cliffs. Moreover, white‐headed langurs exclusively use natural caves on the cliffs as their night‐sleeping sites and sunbathe on bare rocks in winter to reduce the energy in thermoregulation (Huang et al., 2003; Huang & Li, 2005; Li et al., 2011). Foraging on the cliffs is helpful for them to quickly sunbathe after feeding. Limestone‐living primates generally sunbathe on bare rocks to save energy for thermoregulation during the winter (Li et al., 2020, 2021), and they likely do not have to travel from bare rocks/cliffs when young leaves and fruits are not available elsewhere, and adopt an energy‐conservation strategy by reducing travel time when feeding on mature leaves (Li et al., 2019). Additionally, white‐headed langurs inhabiting karst forests maintain a small home‐range size with shorter daily path lengths during the dry months than during the rainy months, constituting an effective energy‐conserving strategy (Huang et al., 2017; Zhou et al., 2011).

White‐headed langurs used hillsides and cliffs more frequently while resting, supporting prediction 2, although they also rested frequently on the hillsides. Primates reduce vigilance behaviors when resting and grooming (Cords, 1995). Consequently, primates prefer using refuges (e.g., tall trees and cliffs) that increase their visibility of predators and reduce predation risk (Cowlishaw, 1997a; Zhou et al., 2013). In limestone forests, cliffs located in upper hills are often vertical rock covered by few vegetation, making the terrestrial predators (such as leopard cats) inaccessible and offering good visibility to the langurs while resting (Zhou et al., 2013). A similar strategy that relies on using the cliffs to reduce predation risk when resting also observed in other primates inhabiting limestone forests, including François's langurs (*Trachypithecus francoisi*) (Chen et al., 2019) and Assamese macaques (Li et al., 2021).

Here, these langurs also spent more time resting on hillsides, even more frequently in the rainy season, probably being linked to food distribution, predation risk, and temperature. As mentioned above, these langurs mainly fed on hillsides and had a folivorous diet. Resting in foraging patches might assist them in digesting fibrous foods (Li et al., 2021). Moreover, hillsides have a dense and high canopy, offering protection against predators (Adams & Teichroeb, 2020). The langurs utilized the flat zones in the dry season more frequently than in the rainy season when resting, possibly because the langurs temporally increase the use frequency of this zone when feeding.

The dense vegetation on hillsides provides shade options for the langurs, particularly during hot and rainy months (Li et al., 2020). Primates frequently stay within the forest shade, avoiding daytime high temperatures and reducing their energetic thermoregulation demands (Duncan & Pillay, 2013; McFarland et al., 2020; Thompson et al., 2016). The temperature of exposed bare rock surfaces rises rapidly during hot summers, while the temperature within the forest is significantly lower (Huang, 2002), supported by our study (Zhang et al., 2021). Consequently, time resting in shaded hillsides is helpful for the langurs to reduce body temperature. Similar behaviors are reported for other sympatric primates [e.g., François' langurs (Li et al., 2020) and Assamese macaques (Li et al., 2021)].

The langurs in both large and small groups primarily foraged on hillsides; however, all groups less frequently foraged on flat zones, partially supporting prediction 3 that the langurs both in large and small groups would mainly forage on hillsides and large groups would expand foraging areas to flat zones for satisfying larger energy needs. As we mentioned previously, hillsides are covered by the most abundant food resources and these langurs could safely harvest foods in these zones. However, the langurs in large groups did not forage more frequently on flat zones than the small groups, despite of having heavier food competition (Zhang et al., 2020). Large groups in current study were closed to a roadside and likely perceived more anthropogenic disturbance from agricultural cultivation and automobile traffic than small groups (Yuan, 2013). As a result, the langurs may infrequently use the flat zones exposed to more human disturbance. The sympatric François' langurs show similar trends in reducing use of bottom zones with heavy human interference (Huang, Wu, et al., 2008). In contrast, the langurs in large groups are more likely to increase their daily path length or decrease their resting time to obtain more foods (Zhang et al., 2020).

The utilization frequency of cliffs by large groups was significantly higher than that by small groups while moving or resting; however, the utilization frequency for hillsides by large groups was smaller than that of small groups when resting. This probably due to predators are mostly a threat to infants (Huang, 2002), and large groups with infants might be more wary of predators. In contrast, groups with more adults/subadults likely suffer less predation presure than groups with more immatures. One limitation of our study is that we do not have data on predator abundance, distribution, and events, due to the topographic conditions.

Our study provides a general pattern of habitat use in the white‐headed langurs. We conclude that the langurs use their habitat differently depending on behaviors, group sizes, spationl distribution of food resources and seasonal variation. We propose this reflects the langurs balancing foraging and thermoregulatory needs with predation risk and human disturbance. Moreover, our results showed that these langurs mostly fed on hillsides and seldom used the flat zones, which may provide conservation management strategies for these langurs. This study suggested that potecting the vegetation on hillsides is important, and vegetation restoration on flat zones is needed. Furthermore, human activities should be reduced or prohibited on flat zones, which would make the langurs increse the use of this zone.

## AUTHOR CONTRIBUTIONS

**Fengyan Liu:** Formal analysis (equal); writing – original draft (equal); writing – review and editing (equal). **Youbang Li:** Data curation (equal); formal analysis (equal); funding acquisition (equal); writing – review and editing (equal). **Kechu Zhang:** Data curation (equal); investigation (equal); writing – review and editing (equal). **Jipeng Liang:** Investigation (equal); writing – review and editing (equal). **Dengpan Nong:** Investigation (equal). **Zhonghao Huang:** Conceptualization (equal); formal analysis (equal); funding acquisition (equal); investigation (equal); methodology (equal); project administration (equal); writing – review and editing (equal).

## CONFLICT OF INTEREST

None declared.

## Data Availability

All data are available in the figshare repository at https://doi.org/10.6084/m9.figshare.19928222.v2.
